# Event-specific qualitative polymerase chain reaction analysis for two T-DNA copies in genetically modified orange *Petunia*

**DOI:** 10.1007/s11240-020-01871-w

**Published:** 2020-06-19

**Authors:** Christian Haselmair-Gosch, Daria Nitarska, Benjamin Walliser, Henryk Flachowsky, Silvija Marinovic, Heidi Halbwirth

**Affiliations:** 1 Institute of Chemical, Environmental and Bioscience Engineering, Technische Universität Wien, Getreidemarkt 9, 1060 Vienna, Austria; 2 Institute for Breeding Research on Fruit Crops, Julius Kühn-Institut, Pillnitzer Platz 3a, 01326 Dresden, Germany

**Keywords:** *Petunia* × *hybrida*, Event-specific transgene detection, Anthocyanin, Orange flower colour, Transgenic plant

## Abstract

In 2017, various orange coloured petunia on the market turned out to be genetically modified (GM) without an official authorization for commercialization. Sequence analysis suggested these undeclared plants most probably originated from a plant transformation experiment performed in the 1980s. For a deeper understanding how GM petunia entered classical breeding programmes worldwide, and whether they originated from a single source or not, we undertook a molecular genetic characterization of the T-DNA integration sites in different GM petunia cultivars and breeding lines. By means of genome walking, we isolated different T-DNA sequences, which are located at the junctions between the T-DNA(s) and the petunia DNA. Based on the results obtained we conclude that there are at least two T-DNA copies of different lengths. This is supported by Southern blot analysis. For T-DNA1, the 3′-junction sequence was isolated, whereas the 5′-junction remained unclear. In contrast, for T-DNA2, the 5′-junction sequence was isolated, whereas the sequence isolated from the 3′-region consists only of T-DNA, but did not include the junction from the T-DNA to the petunia DNA. We developed primers for event-specific PCRs and screened a set of three orange GM petunia cultivars and 126 GM offspring from a commercial breeding program. We show that both T-DNA copies are present in all our tested GM petunia samples, which underpins the assumption of a single transgenic origin of the undeclared GM petunia. Most likely, the two T-DNAs are integrated in close proximity into the petunia genome.

**Electronic supplementary material:**

The online version of this article (10.1007/s11240-020-01871-w) contains supplementary material, which is available to authorized users.

## Key message

The recently escaped genetically modified orange petunia contain at least two T-DNAs, which have the same integration sites in all tested plant samples, indicating a single transgenic origin. Both T-DNAs can be unambiguously identified via event-specific PCRs.

**Electronic supplementary material:**

The online version of this article (10.1007/s11240-020-01871-w) contains supplementary material, which is available to authorized users.

## Introduction

Petunias are economically important balcony and bedding plants, which are available in many different growing shapes and flower colours. However, like some other species (e.g. cyclamen, African violet, *Cymbidium*, *Angelonia*), petunia do not naturally produce orange/bright-red flowers because they lack the ability to synthesize pelargonidin-type anthocyanin pigments (Forkmann and Ruhnau 1987; Gosch et al. 2014; Johnson et al. 1999). This is because of the substrate specificity of petunia dihydroflavonol 4-reductase (DFR), which does not accept the essential pelargonidin-precursor dihydrokaempferol as a substrate during anthocyanin biosynthesis. Starting in the 1980s, transgenic petunia that produce pelargonidin-type anthocyanins were created to achieve orange flower colour, by introducing either a maize *DFR* encoded by the *A1* coding sequence (Elomaa et al. 1995; Meyer et al. 1987), a gerbera *DFR* (Elomaa et al. 1995; Helariutta et al. 1993), a *Calibrachoa DFR* (Chu et al. 2015) or a rose *DFR* (Chu et al. 2015; Tsuda et al. 2004). However, no transgenic orange petunia has been officially commercialized. More than 30 years later, different orange coloured petunia appeared on the market, which were assumed to originate from classical breeding programs and not from biotechnological approaches. In 2017, it turned out that most of the orange petunia contain foreign DNA from maize and plant transformation vectors (Bashandy and Teeri 2017; Haselmair-Gosch et al. 2018). Based on the current information, the transformation construct of the first petunia transformation experiments performed by Meyer et al. (1987) can be assumed to be the source of those undeclared genetically modified (GM) orange petunia (Bashandy and Teeri 2017; Fraiture et al. 2019; Haselmair-Gosch et al. 2018). Subsequently, the cultivars, which were identified as GM, were removed from the market with some exceptions like e.g. Canada (CFIA 2020).

Initially, only PCR screening for transgenic DNA sequences (e.g. for *A1 DFR*, *nptII*, p*35S* promoter DNA elements or construct combinations thereof) was possible for the detection of those GM petunia (Bashandy and Teeri 2017; Haselmair-Gosch et al. 2018). This limits specificity, since such PCR methods do not necessarily discriminate between independent transgenic petunia events, if PCR targets the same genetic elements for e.g. promoters or selection markers (Elomaa et al. 1995; Meyer et al. 1987; Shimada et al. 1999). For petunia, several GM are published, targeting traits like flower colour, early/late flowering, plant morphology, fragrance, longevity or biotic/abiotic stress resistance (for review see Boutigny et al. 2020). Event-specific PCRs are more accurate and allow the analysis of distinct transformation events, since the method is based on the detection of junction sequences between the T-DNA(s) and the host genome, which is unique for each transformation event, even if more than one T-DNA is integrated during the transformation process (Holst-Jensen et al. 2012, 2003). Event-specific PCRs are available for various plants—mainly major crop plants like maize, soybean or canola etc. and are mainly used for screening food for genetic modifications (for review see e.g. Holst-Jensen et al. 2003; Salisu et al. 2017).

Recently, MinION sequencing technology was shown to be suitable for isolating a transgene flanking sequence part of GM petunia, which was identical in the 23 tested cultivars, suggesting a single origin of the GM plants (Fraiture et al. 2019). However, for orange GM petunia, several transgenic lines with a different number of integrated copies of the transgenic sequence were reported from the initial transformation experiment (Linn et al. 1990) but it is still unclear, which of these could be the source of the undeclared orange GM petunia.

For a deeper understanding how GM petunia entered classical breeding programmes worldwide and whether they originated from a single source or not, we aimed for the molecular genetic characterization of the T-DNA integration sites in different GM petunia cultivars and breeding lines.

## Materials and methods

### Chemicals

Primers for Southern blot probe amplification were synthesized by Eurofins (Germany). All other oligonucleotide primers were synthesized by Sigma-Aldrich (Austria). dNTPs were ordered from Fermentas (Germany). Standard chemicals (e.g. Tris, solvents, agarose, CTAB) were purchased from Sigma-Aldrich (Austria), Merck (Austria) or VWR (Austria). Ultrapure water was used (Ultrapure Direct-Q 3 UV equipped with a Millipak Express 20 filter; Merck, Austria).

### Plant samples

Young flowers or young leaves of different *Petunia* × *hybrida* cultivars or breeding lines were used. The GM cultivars were ‘Salmon Ray’ (Danziger, Moshav Mishmar Hashiva, Israel), ‘Viva Orange’ (Florensis, Ambacht, The Netherlands) and ‘Electric Orange’ (Selecta One, Germany). Non-GM control plants of *Petunia* cv. ‘Baby Doll’ were obtained from Selecta One (Germany), those of cv. ‘Blackberry’ were purchased from Austrosaat (Vienna, Austria). Moreover we used 154 individual crossings of a commercial breeding program, which involved 179 different parental lines in various combinations. From those 154 crossings, 126 were GM and 28 were not. GM was tested by means of PCR with two primer pairs targeting for fragments of the *A1 DFR* coding sequence of maize and the *nptII* gene as described earlier (Haselmair-Gosch et al. 2018). Plant material was harvested, shock-frozen and kept at − 80 °C until analysis. Plant material was ground in liquid nitrogen and genomic DNA was isolated either with the Invisorb Spin Plant Mini Kit (Invitek Molecular, Germany) or the CTAB method (Lipp et al. 1999).

### Genome walking for T-DNA integration site analysis

The GenomeWalker Universal Kit (Clontech, Takara Bio Inc., USA) was used according to the manufacturer’s instructions to determine the 5′- and 3′-junction sequences spanning the T-DNA and the adjacent plant DNA. In brief, four GenomeWalker DNA libraries were constructed with genomic DNA of cv. ‘Viva Orange’ using the restriction enzymes *Dra*I, *Eco*RV, *Pvu*II and *Stu*I with subsequent GenomeWalker adaptor ligation. Three gene specific (nested) reverse and forward primers were designed close to the 5′- and 3′-end of the NCBI genbank sequences MF521566 (Haselmair-Gosch et al. 2018) and KY964325 (Bashandy and Teeri 2017), representing the transgene construct sequence of orange GM petunia. Adaptor primers AP1 and AP2 were provided by the GenomeWalker Kit. All primer sequences are listed in Table 1. Genome walking PCR reactions were performed in a Mastercycler ep Gradient PCR device (Eppendorf, Germany) in a final volume of 20 µl consisting of: 1 × Go*Taq* Green reaction buffer, 0.4 µl dNTPs (10 mM), 1 µl forward and reverse primer each (10 µM), 1 µl GenomeWalker library DNA and 0.2 µl DNA polymerase (5 U/µl, Go*Taq* DNA Polymerase Kit, Promega, Germany). In the primary PCR the primers p35S-R6 (5′-junction) or gm-ocs-F1 (3′-junction) were used in combination with the genome walker primer AP1 and the following cycling conditions: 94 °C 1 min; 40 cycles (94 °C 30 s, 67 °C 30 s, 72 °C 2 min) and a final extension at 72 °C for 7 min. For the secondary PCR the primers p35S-R5 (5′-junction) or gm-ocs-F2 (3′-junction) were used in combination with the genome walker primer AP2 (double nested) at the same cycling conditions as for the primary PCR. 1 µl of a 1:10 dilution of the primary PCR reaction was used as template despite no amplification products being detected by agarose gel analysis of the primary PCR reaction. Single amplification products of the secondary PCR were cut out and eluted after 1% agarose gel electrophoresis and used in a 1:10 dilution as template (1 µl) for the tertiary PCR. Tertiary PCR was performed with the primers p35S-R4 (5′-junction) or gm-ocs-F3 (3′-junction) in combination with the genome walker primer AP2 (single nested), again at the same cycling conditions as mentioned for the primary PCR. Amplification products were cut and eluted after 1% agarose gel electrophoresis and used for direct PCR sequencing, which was performed by Microsynth (Switzerland).

**Table 1:** Oligonucleotide primers

Primer name	Sequence (5′ > 3′)	Application
AP1	GTAATACGACTCACTATAGGGC	Genome walking (adaptor primer)
AP2	ACTATAGGGCACGCGTGGT	Genome walking (adaptor primer)
DFR_A1a_Fwd	GGAAGACGAAGCCATTGAT	Southern blot analysis (*A1* probe generation)
DFR_A1a_Rev	GTGCGAGGAGCAAACGAA	Southern blot analysis (*A1* probe generation)
gm-ocs-F1	GGTTGGGCTTCGGAATCGTTTTCCG	3′-genome walking (gene specific primer)
gm-ocs-F2	GAGATATGCGAGACGCCTATGATCGCAT	3′-genome walking (gene specific primer)
gm-ocs-F3	CCTGAGCATGTGTAGCTCAGATCCTTAC	3′-genome walking (gene specific primer)
gm-P-F3	CTCCCACAGAGATTCCAAAGGCAGTAGAC	Forward primer specific for Pet_5′T-DNA2 amplification
gm-P-R6	GTCATCAAAGGCTTGAGATGTGAACTCACC	Reverse primer specific for 3′T-DNA1_Pet amplification
nptII_F	ACAAGATGGATTGCACGCAGG	Southern blot analysis (*nptII* probe generation)
nptII_R	AACTCGTCAAGAAGGCGATAG	Southern blot analysis (*nptII* probe generation)
ocs-l-R1	GGGATCGAGCCCCTGCTGAG	Reverse primer specific for 3′T-DNA2 amplification
p35S-R4	ATCAGTTGGGTGCACGAGTGGGTTACAT	5′-genome walking (gene specific primer) and reverse primer specific for Pet_5′T-DNA2 amplification
p35S-R5	ACTTTTCGGGGAAATGTGCGCGGAACC	5′-genome walking (gene specific primer)
p35S-R6	AAGACGAAAGGGCCTCGTGATACGCCTATT	5′-genome walking (gene specific primer)
Pet-DFR-F1	TCACTTCATCTGCTGGAACTCTCGATG	Forward primer specific for petunia dihydroflavonol 4-reductase
Pet-DFR-R	GCCTCACAAAGATCATCCAAATGCACATAT	Reverse primer specific for petunia dihydroflavonol 4-reductase
rc-ocs-k-R2	CTGATTGTACCCTACTACTTATATGTACAA	Forward primer for 3′T-DNA1_Pet and 3′T-DNA2 amplification

### Event-specific and element-specific qualitative PCR

**Fig. 1:**
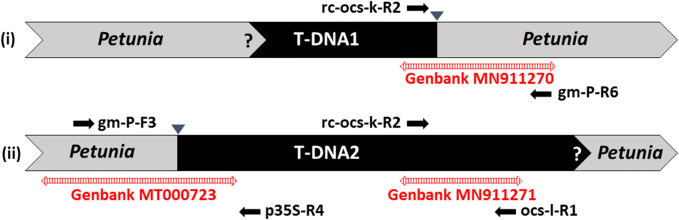
Schematic overview of the situation at the genomic integration sites of (i) T-DNA1 and (ii) T-DNA2. Single arrows in black show the location and direction of primers for specific detection of the two T-DNAs. Double arrows in red indicate sequence sections identified by means of genome walking during this study. Question marks represent the unknown junctions and triangles the identified junctions from T-DNAs to petunia DNA. The drawing does not reflect exact size relations

Primers were designed based upon the sequences obtained by genome walking and are listed in Table 1 and shown in Fig. 1. Event-specific PCR primers for T-DNA1 were rc-ocs-k-R2 and gm-P-R6 (60 °C PCR annealing temperature) and those for T-DNA2 were gm-P-F3 and p35S-R4 (65 °C PCR annealing temperature), which allow the amplification of the event-specific junctions between the T-DNAs and the petunia DNA. Additionally, element-specific PCR primers for the *octopine synthase* gene terminator sequence (t*OCS*) of T-DNA2 were designed (rc-ocs-k-R2, ocs-l-R1, 60 °C PCR annealing temperature). To verify the integrity of petunia DNA samples, primers specific for a petunia *DFR* gene were used (Haselmair-Gosch et al. 2018), resulting in an amplicon of 565 bp. Water and DNA of non-GM petunia were used as negative controls. The PCR reactions were performed in a final volume of 20 µl consisting of: 1 × Go*Taq* Green reaction buffer, 0.4 µl dNTPs (10 mM), 1 µl forward and reverse primer each (10 µM), 1 µl DNA (50 ng/µl) and 0.2 µl DNA polymerase (5 U/µl, Go*Taq* DNA Polymerase Kit, Promega, Germany). The following cycling conditions were used in a Mastercycler ep Gradient PCR device (Eppendorf, Germany): 98 °C 2 min; 40 cycles (98 °C 30 s, 50 s specific annealing temperature, 72 °C 40 s) and a final extension at 72 °C for 10 min. PCR products were separated on a 2% agarose gel and 1 × buffer TAE and stained with SERVA DNA Stain Clear G (SERVA Electrophoresis GmbH, Germany). The 1 kb Plus DNA ladder (NewEngland Biolabs, Austria) was used as a molecular size standard. Gels were scanned and evaluated with an Amersham Typhoon 5 Biomolecular Imager (GE Healthcare Bio-Sciences Corp., Austria).

### Southern blot analysis

Detection of integrated T-DNA copy numbers was performed by Southern hybridization. 10 µg DNA of the orange GM petunia cvs. ‘Viva Orange’, ‘Electric Orange’ and ‘Salmon Ray’ as well as the wild type cv. ‘Baby Doll’ (used as negative control) were incubated with 100 units of *Eco*81I or *Bsp*OI (Thermo Fisher Scientific, Germany) at 37 °C over night. The cleaved DNA was separated on a 0.8% agarose gel and transferred onto a positively charged nylon membrane (Roche Diagnostics, Germany). Two PCR amplified, digoxigenin-labeled probes amplified either from the *A1 DFR* coding sequence (probe length: 641 bp) or the *nptII* marker gene (probe length: 780 bp) sequences were generated (primers listed in Table 1) using the PCR DIG Probe synthesis Kit (Roche Diagnostics, Germany) and used for hybridization. Hybridization and detection were performed using the ECF-Random-Prime-Labeling and Detection Kit (GE Healthcare Amersham™ Biosciences, UK) according to the manufacturer’s manual.

## Results

### T-DNAs integration site analysis

**Fig. 2:**
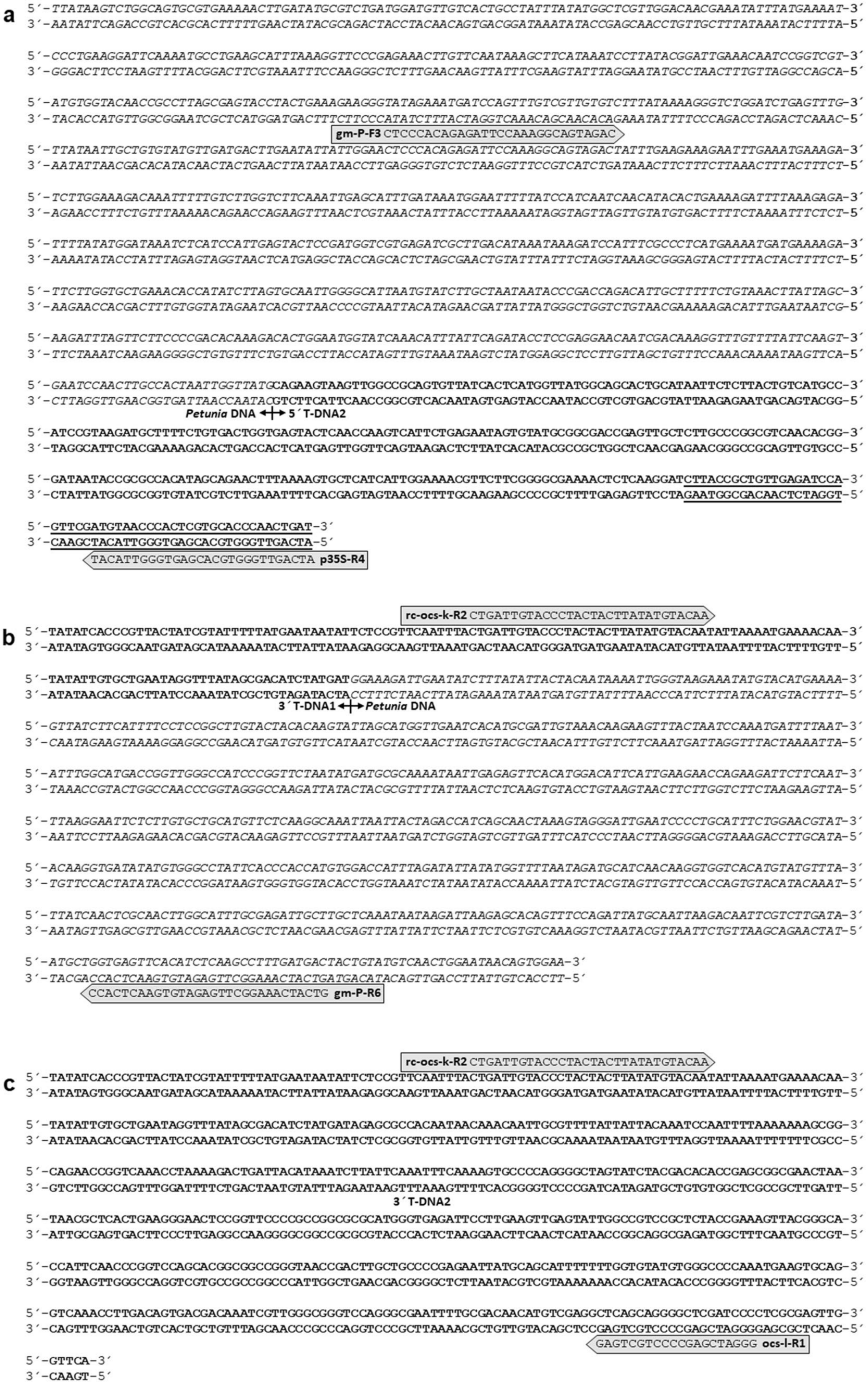
Sequences of **a** Pet_5′T-DNA2 (1078 bp, accession No. MT000723) plus underlined sequence originating from accession No. KY964325 (Bashandy and Teeri 2017), **b** 3′T-DNA1_Pet (765 bp, accession No. MN911270) and **c** 3′T-DNA2 (605 bp, accession No. MN911271) obtained by genome walking. PCR primers derived thereof are given. Bold letters indicate T-DNA sequences, italic letters are petunia DNA

Genome walking technology was used to isolate the T-DNA junction sequences between the known T-DNA sequences MF521566 and KY964325, which are available at the database of the National Center for Biotechnology Information (NCBI), and the petunia genome in the orange GM petunia cv. ‘Viva Orange’. Originally, only the presence of a single T-DNA in the genome was expected. After tertiary PCRs using the four GenomeWalker libraries as template, three putative T-DNA junction sequences were obtained from two T-DNAs inserted into the petunia genome (Fig. 1). These sequences were designated as Pet_5′T-DNA2, 3′T-DNA1_Pet and 3′T-DNA2 (Fig. 2).

For T-DNA2 only the 5′ junction sequence could be isolated. This 5′T-DNA2 sequence together with the upstream petunia DNA was obtained from the GenomeWalker library digested with *Eco*RV. The 1,078 bp sequence was deposited in the NCBI database (accession No. MT000723) and comprises identically a recently isolated junction sequence of 402 bp of GM petunia (Fraiture et al. 2019). The entire sequence of the Pet_5′T-DNA2 fragment consists of 828 bp petunia DNA followed by 250 bp of a *β*-lactamase gene (*bla*) in antisense direction. Six frame translation of the petunia DNA upstream of 5′T-DNA2 resulted in a partial open reading frame of 275 codons from a protein with highest homology to retrotransposon polyproteins found in e.g. *Coffea* (AQY61297) or *Vitis* (RVW93168).

Searching for 3′T-DNA junction sequences, two different sequences with a length of 765 bp and 605 bp, respectively, were found. The first sequence was obtained from the GenomeWalker library digested with *Stu*I, whereas the second sequence was obtained from the library digested with *Pvu*II. Both sequences were deposited in the NCBI database under the numbers MN911270 for 3′T-DNA1_Pet and MN911271 for 3′T-DNA2. The sequence 3′T-DNA1 contains 138 bp, which are identical to the corresponding section of the longer 3′T-DNA2, which has a total size of 605 bp. 3′T-DNA2 is identical to commonly used plant transformation vector sequences (e.g. accession No. JQ974028) and consists of mainly the *octopine synthase* terminator. Six frame translation of the petunia DNA (627 bp) downstream of 3′T-DNA1 resulted in a partial open reading frame of 209 codons, again with highest homology to retrotransposon polyproteins as described before.

By performing PCRs with forward primers designed against the petunia DNA upstream of 5′T-DNA2 and reverse primers specific to 3′T-DNA2 we found, that sequence 5′T-DNA2 belongs to sequence 3′T-DNA2 of the same T-DNA (Supplemental Figure S1).

The petunia DNAs upstream of 5′T-DNA2 and downstream of 3′T-DNA1 were mapped to publically available genome sequences of *P. inflata* and *P. axillaris* (www.solgenomics.net), which represent the two ancestors of *P.* × *hybrida* (Bombarely et al. 2016; Fernandez-Pozo et al. 2015). Independent mapping of the two petunia DNAs revealed highly similar sequences in several of the same scaffolds but in a different BLAST results order. Interestingly, petunia DNA of 3′T-DNA1_Pet is located in silico in the genome closely upstream (1258 bp distance) of Pet_5′T-DNA2, suggesting a tandem insertion of T-DNA1 and T-DNA2, with a petunia DNA spacer piece of all together 2,224 bp (138 bp + 1258 bp + 828 bp) of petunia DNA in between (e.g. *P. axillaris* v1.6.2 genome Scaffold Peaxi162Scf00714). However, by means of PCR with different primer combinations (e.g. 3′T-DNA1 specific forward and 5′T-DNA2 specific reverse primers) such a tandem integration could not be verified.

The 3′T-DNA2, which was isolated by genome walking did not include the junction from the T-DNA to the petunia DNA, because *Pvu*II, which was successful for GenomeWalker library generation cuts within the sequence of 3′T-DNA2. Using the other three libraries, where the DNA was cut with either *Dra*I, *Eco*RV or *Stu*I, respectively, failed to isolate the T-DNA flanking petunia sequence.

For T-DNA1, no 5′ junction sequence could be isolated by means of two independent 5′ genome walking experiments using the primers specific for the CaMV 35S promoter p*35S*. In addition, another approach was used to identify the missing junctions of T-DNA1 and T-DNA2: Therefore, several PCR primers were designed based on sequences adjacent to the putative insertion regions, which were determined within the genomes from *P. axillaris* and *P. inflata* by using the isolated petunia DNA downstream of T-DNA1 or upstream of T-DNA2. Those primers were used together with primers specific for T-DNA1 or T-DNA2, respectively. Unfortunately, this strategy has so far failed to amplify the missing junctions (data not shown).

### Southern blot analysis

**Fig. 3:**
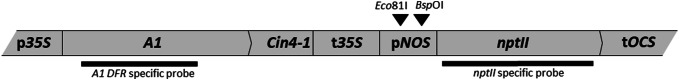
Schematic representation of the position of the restriction sites *Eco*81I and *Bsp*OI and the Southern probes for *A1 DFR* and *nptII* located on the transgenic insert found in orange GM petunia (accession No. MF521566 (Haselmair-Gosch et al. 2018)). p*35S*, promoter sequence of the *35S* Cauliflower mosaic virus gene; *A1*, coding sequence of the *A1 DFR* gene; *Cin4-1*, partial *Cin4-1* transposable element present in type 2 allele of *A1 DFR* (Schwarz-Sommer et al. 1987a, b); t*35S*, terminator sequence of the *35S* Cauliflower mosaic virus gene; p*NOS*, promoter sequence of the *nopaline synthase* gene; *nptII*, coding sequence of the *neomycine phosphotransferase II* selectable marker gene; t*OCS*, terminator sequence of the *octopine synthase* gene. The drawing does not reflect exact size relations

**Fig. 4:**
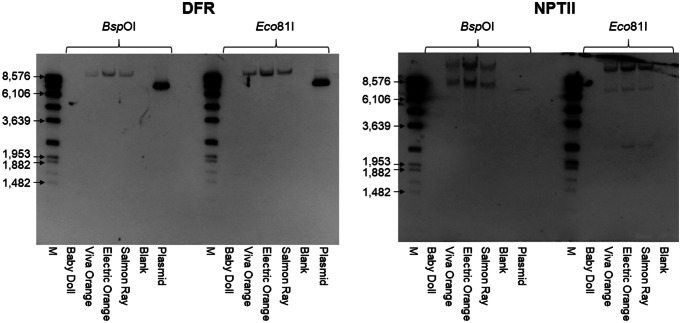
Southern blot analysis of orange GM petunia cultivars ‘Viva Orange’, ‘Electric Orange’ and ‘Salmon Ray’ with probes for the *A1 DFR* coding sequence (left) and the *nptII* gene (right). Wild type cv. ‘Baby Doll’ was used as non-transgenic negative control. A plasmid harbouring the *A1 DFR* coding sequence was used as positive control for *DFR* probe. The restriction endonucleases *Bsp*OI and *Eco*81I were used. M, molecular size standard (DIG Molecular Weight Marker VII, Roche Diagnostics, Germany); Blank, H_2_O used as negative control. Selected fragments of the size marker are labelled with fragment lengths in bp for better orientation. Smaller fragments of the size standard are not visible

Southern blot analysis was performed with three orange GM petunia cultivars and one wild type cultivar used as negative control. The restriction enzymes *Eco*81I and *Bsp*O1 and probes specific to *A1 DFR* and *nptII* sequences were used (Fig. 3). Hybridization revealed for all GM cultivars only one copy of *A1 DFR*, but two to three copies of *nptII* (Fig. 4).

### Event-specific and element-specific qualitative PCR

**Fig. 5:**
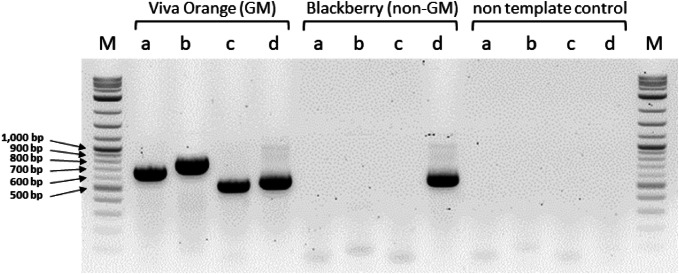
PCR evaluation of petunia DNA with primers specific for 3′T-DNA1_Pet, Pet_5′T-DNA2 and 3′T-DNA2. A 2% agarose gel was used. a, primers rc-ocs-k-R2 and gm-P-R6 for 3′T-DNA1_Pet (682 bp amplicon); b, primers gm-P-F3 and p35S-R4 for Pet_5′T-DNA2 (791 bp amplicon); c, primers rc-ocs-k-R2 and ocs-l-R1 for 3′T-DNA2 (536 bp amplicon); d, primers Pet-DFR-F1 and Pet-DFR-R for a partial sequence of the petunia *DFR* (565 bp amplicon); GM, genetically modified; M, molecular size standard 2-Log DNA Ladder

Event-specific and element-specific qualitative PCR was developed and optimized by using DNA of cv. ‘Viva Orange’ as template, for which the specificity of the products was confirmed by agarose gel electrophoresis and sequencing by Microsynth (Austria). The primers (Table 1 and Fig. 2) were designed upon the sequences obtained by genome walking and showed in silico no homology to any sequence present in the publicly available genome data of *Petunia inflata* or *P. axillaris* (Bombarely et al. 2016; Fernandez-Pozo et al. 2015). Event-specific primers rc-ocs-k-R2 and gm-P-R6 were used to amplify a 682 bp sequence of the junction of 3′T-DNA1_Pet. For the junction of Pet_5′T-DNA2 the primers gm-P-F3 and p35S-R4 were used, with the expected amplicon size of 791 bp. Element-specific PCR for the rear part of T-DNA2 was done with primers rc-ocs-k-R2 and ocs-l-R1, with the expected amplicon size of 536 bp. Figure 5 shows representative results of positive and negative samples as well as negative controls. We screened the orange GM petunia cultivars ‘Salmon Ray’, ‘Viva Orange’, ‘Electric Orange’ and 126 GM offsprings from individual crossings of a commercial breeding program. As non-GM controls, 28 offsprings and the cultivar ‘Blackberry’ were used. All GM samples showed amplification products at the expected length, which are presented exemplarily in Fig. 5.

## Discussion

In 1986, at the Max Planck Institute for Plant Breeding Research in Cologne, Germany, the first modification of a flower’s colour by a transgenic approach was achieved (Meyer et al. 1987). Doubtless, this was a milestone for flower colour research and subsequently enabled a deeper understanding of e.g. epigenetic changes via DNA methylation, gene copy numbers or the T-DNA integration site, etc. (Linn et al. 1990; Meyer et al. 1992; Pröls and Meyer 1992). Orange GM petunia of those experiments were the first GM plants, which were released into the field in Germany and attracted attention far beyond the horticultural community. Although the orange GM petunia has never been officially commercialized, in 2017 different orange coloured petunia were identified on the market without GM declaration. There are several documented cases where un-authorized GM plant species or products thereof unintentionally emerged on the market or in the food chain via different ways (Holst-Jensen et al. 2012). Based on the current sequence information, the transformation construct of the first petunia transformation experiments performed by Meyer et al. (1987) can be assumed to be the source of those undeclared orange GM petunia (Bashandy and Teeri 2017; Fraiture et al. 2019; Haselmair-Gosch et al. 2018). To understand, how GM petunia could enter classical breeding programmes worldwide and whether they originated from a single source or not, we aimed for the molecular genetic characterization of the T-DNA integration sites in different GM petunia cultivars and breeding lines. By means of genome walking techniques, we isolated three putative T-DNA junction sequences from two T-DNAs (T-DNA1, T-DNA2) inserted into the petunia genome.

For T-DNA2 only the 5′ junction sequence could be isolated, which was designated as Pet_5′T-DNA2. For the rear part of T-DNA2, only a T-DNA specific sequence was isolated (designated as 3′T-DNA2), whereas the junction and adjacent petunia DNA remained unclear. Most probably, because *Pvu*II was used during GenomeWalker library construction, which was found to cut within T-DNA2 but not in the petunia DNA flanking this T-DNA sequence.

In contrast, the 3′ junction sequence of T-DNA1 (designated as 3′T-DNA1_Pet) was obtained from the GenomeWalker library digested with *Stu*I, which cuts within the petunia genome downstream of T-DNA1. However, for T-DNA1, no 5′ junction sequence could be isolated by means of two independent 5′ genome walking experiments using the primers specific for the CaMV *35S* promoter p*35S*. We assume that T-DNA1 could be truncated at the front part, and is therefore incapable of binding p*35S* specific primers during genome walking, compared to T-DNA2. This is in accordance with the results of Fraiture et al. (Fraiture et al. 2019), who used a p*35S* specific target for isolating 5′ T-DNA flanking sequences and could also only identify one T-DNA. The hypothesis of T-DNA1 being truncated in the front part is underpinned by our Southern hybridization results, where only one copy of the *A1 DFR* coding sequence (referring to T-DNA2) was found, whereas two to three copies of the *nptII* gene were detected. Most likely, T-DNA1 is truncated at the 5′-side somewhere before or within the *A1 DFR* sequence to such an extent that the *A1 DFR* probe is not able to bind for a Southern blot based detection. Southern hybridization was done with a small selection of three orange GM petunia cultivars. Despite those cultivars originate from three independent companies from different countries it cannot be excluded that other—currently undetected—T-DNAs are present in GM petunia. However, in general, uniform and stable orange colouration was commonly observed only when 1–2 intact copies of the *A1* coding sequence were integrated into the genome (Linn et al. 1990; Tsuda et al. 2004). From the initial transformation experiment (Meyer et al. 1987), several lines with a different number of integrated copies of the transgenic sequence, which ranged mainly from 1 to 8 copies, are known (Linn et al. 1990). Some of them, like No. 235/1-15 or No. 235/1-17, which both have only one intact *A1 DFR* coding sequence copy integrated, were later used as donors in classical breeding programs, to obtain petunia with improved phenotypical expression of the orange flower colour (Oud et al. 1995). Those lines could also be the source of the illegally marketed GM-petunia.

For the two T-DNAs that were isolated in the present study, we developed event-specific PCRs for the junction sequences of 3′T-DNA1_Pet and Pet_5′T-DNA and an element-specific PCR for 3′T-DNA2, targeting the *octopine synthase* gene terminator t*OCS*. Since event-specific PCRs are based on the detection of junction sequences between the T-DNA(s) and the host genome, they allow the analysis of distinct transformation events, even if more than one identical copy is integrated. We screened the orange GM petunia cultivars ‘Salmon Ray’, ‘Viva Orange’, ‘Electric Orange’ and 126 GM offspring from individual crossings of a commercial breeding program by PCR. All GM samples have both T-DNAs integrated at the same location in the host genome, which underpins the assumption of a single transgenic line as source of the undeclared GM petunia. Since previous results have shown that the presence of the *A1 DFR* coding sequence in GM petunia does not necessarily result in orange phenotypes in a common biochemical petunia background (Haselmair-Gosch et al. 2018), also non-orange petunia could have a concealed transgenic status, which can also be determined with our PCR protocols.

We mapped our two identified petunia DNA sequences adjacent to 5′T-DNA2 and 3′-TDNA1 by using the publically available genome sequences of petunia (Bombarely et al. 2016; Fernandez-Pozo et al. 2015) and identified several scaffolds, indicating an insertion into a repetitive genome region. In silico, petunia DNA of 3′T-DNA1_Pet is located in the genome closely upstream of Pet_5′T-DNA2, suggesting a tandem insertion of T-DNA1 and T-DNA2, which could not be verified by means of PCR with different primer combinations. This could be because of possible insertional effects like genomic rearrangements, duplication or deletion events, which may have occurred during the transfer of the T-DNAs as a commonly known phenomenon (Schnell et al. 2015), or the assumption that the T-DNAs are inserted into a region of repetitive sequences. Nevertheless, at least the insertion in close proximity on the same chromosome can be supposed, since screening of our 129 GM petunia from individual crossings with event-specific PCRs showed 100% co-segregation of both T-DNAs.

## Electronic supplementary material

Below is the link to the electronic supplementary material.

Supplementary file1 (PDF 393 kb)

## Data Availability

All data generated or analyzed during this study are included in this published article and its supplementary information file. Primary datasets are available from the corresponding author on reasonable request.
