# Supplementary material for: Event-specific qualitative polymerase chain reaction analysis for two T-DNA copies in genetically modified orange Petunia
Source: Plant Cell Tissue Organ Cult. 2020 Jun 19;142(2):415–24. doi: 10.1007/s11240-020-01871-w (PMC7359168; doi:10.1007/s11240-020-01871-w)
Supplement: Supplementary file 1 — Supplementary file1 (PDF 393 kb) [file 11240_2020_1871_MOESM1_ESM.pdf]

Suppl. Fig S1 Mapping of the sequences MT000723 (5´ genome walking) and MN911271 (3´ genome walking) to T-DNA2 by means of PCR

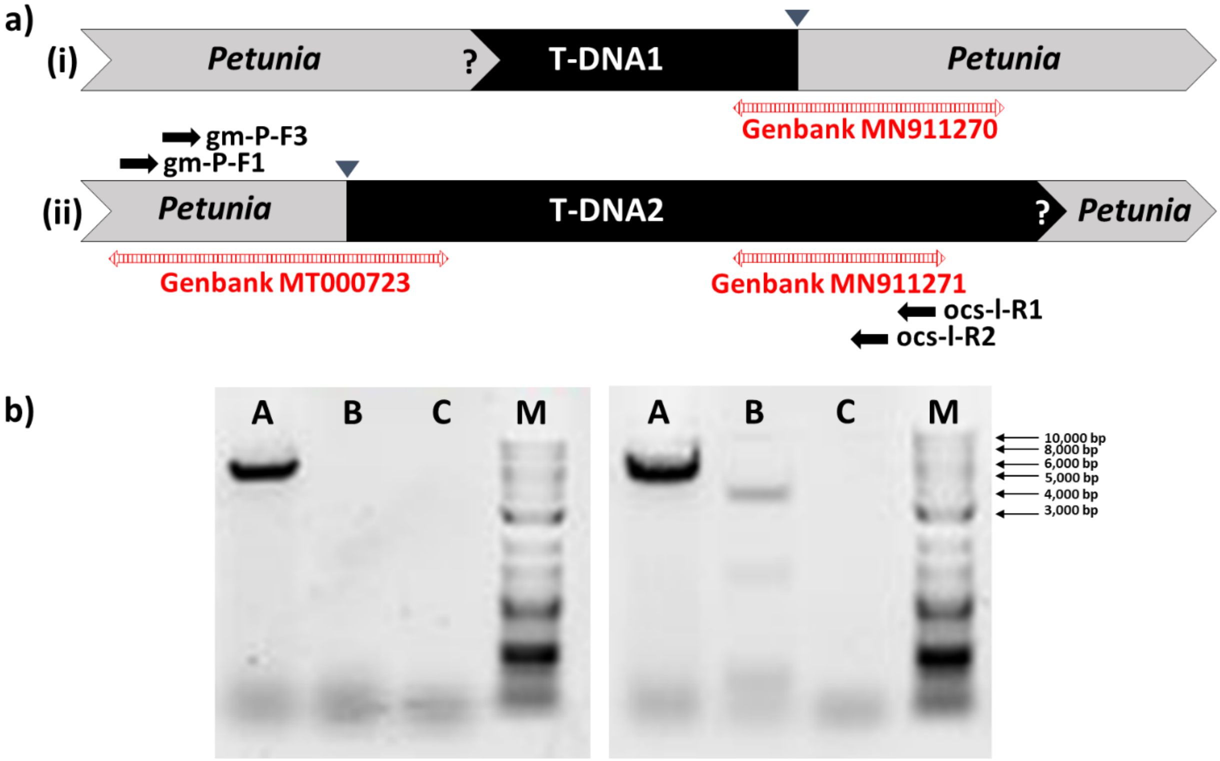

a) Schematic overview of the situation at the genomic integration sites of (i) T-DNA1 and (ii) T-DNA2. Single arrows in black show the location and direction of primers. Double arrows in red indicate sequence sections identified by means of genome walking during this study. Question marks represent the unknown junctions and triangles the identified junctions from T-DNAs to petunia DNA. The drawing does not reflect exact size relations.

b) 1% agarose gel evaluation of PCR products. Two different primer combinations were used to show that the 5´ junction (accession No. MT000723) obtained by genome walking belongs to 3´-T-DNA2: left: gm-P-F1 (GTCACTGCCTATTTATATGGCTCGTTGGAC) + ocs-l-R1 (GGGATCGAGCCCCTGCTGAG) (expected amplicon size of 5,627 bp). right: gm-P-F3 (CTCCCACAGAGATTCCAAAGGCAGTAGAC) + ocs-l-R2 (GTTGTCGCAAAATTCGCCCTGGAC) (expected amplicon size of 5,306 bp). A, GM petunia cv. 'Viva Orange'; B, wild type petunia cv. 'Blackberry'; C, non template control (water); M, molecular size standard 2-Log DNA Ladder
